# Cataract surgery with implantation of a high‐add intraocular lens LENTIS® MAX LS‐313 MF80 in end‐stage, age‐related macular degeneration: A case report of magnifying surgery

**DOI:** 10.1002/ccr3.1912

**Published:** 2018-11-13

**Authors:** Andreas F. Borkenstein, Eva‐Maria Borkenstein

**Affiliations:** ^1^ Borkenstein & Borkenstein, Private Practice Privatklinik der Kreuzschwestern Graz Graz Austria

**Keywords:** AMD, ARMD, cataract surgery, high‐add intraocular lens, magnifying surgery, MAGS

## Abstract

A new treatment option for cataracts and advanced ARMD: the simultaneous intravitreal injection of anti‐vascular endothelial growth factor and implantation of a high‐add IOL. We believe major opportunities arise from a new subcategory in cataract surgery—called magnifying surgery (MAGS). Our case proves the potential of this brand‐new technology.

## 1 INTRODUCTION

Simultaneous presence of cataract and age‐related macular degeneration can cause loss of distance vision and impairment of the patients' quality of life. The simultaneous intravitreal injection of anti‐VEGF (vascular endothelial growth factor) agents, and implantation of a high‐add IOL (intraocular lens) is a new treatment option, called magnifying surgery (MAGS).

Age‐related macular degeneration (ARMD) is one of the most disabling diseases for visual quality and the most common cause of near vision loss among patients older than 65 years in industrialized countries.^1^ The final stage of ARMD is characterized by central scotomas, which makes it impossible to read or to bring near objects into focus. On the contrary, the peripheral vision is usually less affected. ARMD is responsible for nearly 10% of all blindness worldwide; furthermore, the most common cause of blindness in developed countries, particularly in people older than 65 years.^2^ The age, the cognitive ability and adaptability plays another important role in overall assessment. Regarding to a systematic review and meta‐analysis, 8.7% of the worldwide population (2017) has ARMD, and the projected number of people with ARMD is around 196 million in 2020, increasing to 288 million in 2040.^3^, ^4^ The disease is more prevalent in Europe than in Asia and increases rapidly after age of 75 years.^2^ There have been significant advances in the effective therapy of wet (exudative) ARMD with intravitreal injections of antiangiogenetic substances.^5^, ^6^, ^7^, ^8^, ^9^

In many cases, patients suffer not only from dry or wet ARMD but have developed clinically significant cataracts additionally. The risk of cataract increases with each decade of life starting around age 40. Cataracts have a prevalence of about 40% at the age of 70 years, increasing to 70% among the 80‐year‐olds.

Although there have been many studies and research on ARMD around the world, there is no clear guidance on the treatment of ARMD accompanied by cataract formation. Objective tests and standard visual acuity examinations are challenging and limited due to the central scotomas. One of the goals of this case report is to change conventional thinking with regard to the treatment of patients with advanced ARMD and cataracts. We want to present the first implantation of a brand‐new high‐add intraocular lens in Austria and one of the first procedures performed worldwide. We call this new treatment option “magnifying surgery” (MAGS).

## 2 CASE REPORT

**Figure 1:**
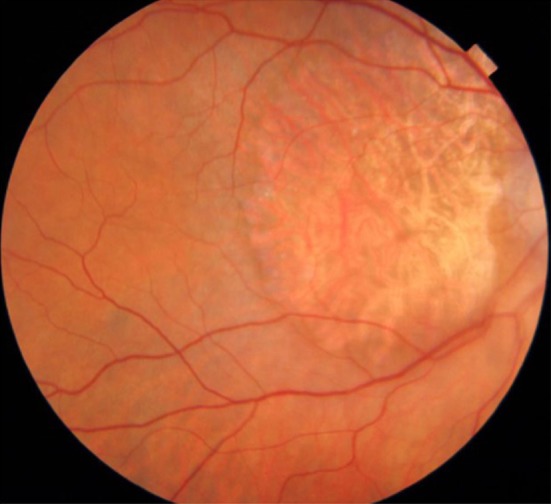
Age‐related macular degeneration choroidal neovascularization (CNV) with cystic edema

A female 86‐year‐old patient presented at our clinic for a second opinion regarding her ocular symptoms. She was already diagnosed with late‐stage macular degeneration in both eyes, progressed cataract formation and pseudoexfoliation syndrome about 6 years ago (Figure 1). She complained about her significantly decreased visual acuity at near and far distance. The measurement of best visual acuity using a semiquantitative scale resulted in “counting fingers” (right eye) and “hand motion” (left eye) at 30 cm distance. The slit lamp examination showed the typical pseudoexfoliation (PXF) disk over the anterior capsule in both eyes. The abnormal white, grayish granular flakes were visible on the anterior capsule and in the trabecular meshwork. The pupils reacted very little to dilating eye drops, caused by posterior synechiae. Any kind of previous intraocular inflammation (uveitis) was assumed but not confirmed. The analysis of the lens showed nuclear and cortical cataracts (NC6) according to the lens opacity classification system (LOCS III).^10^ Intraocular pressure was fluctuating between 18‐26 mm Hg without any therapy during the last months. Fundoscopy revealed a late‐stage macular degeneration with large geographic atrophy in the right eye. A subretinal hemorrhage with cystic edema was found in the left eye. Multiple intravitreal injections in both eyes had been performed at another clinic about 4‐5 years ago. Clinical reports of that time were not available. The intravitreal therapy of the patient was interrupted about 2 years ago. Since then, there have not been any control examinations and further therapies.

Intravitreal injection therapy in the left eye was reinitiated and we suggested to perform cataract surgery to improve visual acuity. About 4 months later, after two Bevacizumab injections in an interval of 4 weeks, the anti‐VEGF therapy was stopped by another clinic due to lack of effectivity and the patient was discouraged regarding cataract surgery.

When the patient presented again at our clinic, we reassessed all clinical findings, repeated OCT examinations of the macula and performed a slit lamp examination of the lens. We found a massive cystic edema with subretinal hemorrhage and retinal pigment atrophy in the left eye and small cystoid spaces (edema) without any new hemorrhage but geographic atrophy in the right eye. Cataracts had advanced in both eyes during the last months, positively correlated to the subjective increase of foggy, gray vision in the periphery, resulting in a significant impairment of the patient's quality of life and ability to perform daily tasks.

**Figure 2:**
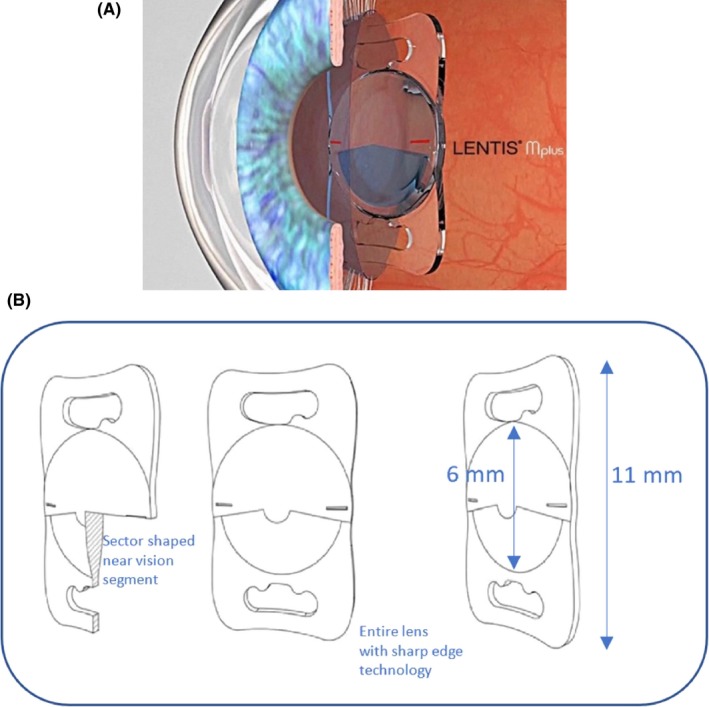
A, Lentis LS‐313 MF80 (Oculentis). B, Specifications of Lentis LS‐313 MF80 with sector‐shaped near vision segment and sharp edges (optic and haptic)

In 2015, we decided to perform phacoemulsification with implantation of a new high‐add intraocular lens (LENTIS® MAX, LS‐313 MF80; Oculentis, Berlin, Germany) in the right eye (Figure 2A,B) and implantation of a monofocal, hydrophobic, acrylic, aspheric IOL (Tecnis PCB00; Johnson & Johnson Vision, Jacksonville, FL, USA), targeting emmetropia in the left eye. The magnifying IOL enables a 3× magnification at 15 cm distance. Cataract surgery using a Malyugin ring (6.25) was done without any complications. Due to the pseudoexfoliation syndrome with small pupils and the advanced cataract formation, the surgery was challenging. Phaco energy was applied as low as possible using a chop technique. The eye was refilled several times during phacoemulsification with cohesive and dispersive ophthalmic viscoelastic devices (softshell technique) to avoid any damage of the macula and the lens capsule/zonular.^11^ At the end of each surgery, a standard intravitreal injection (IVOM) with Aflibercept (Eylea, 40 mg/mL; Bayer, Leverkusen, Germany) was performed.

The postoperative course was uneventful. The postoperative local therapy scheme included cortisone and nonsteroidal anti‐inflammatory drugs. In addition, a carbonic‐anhydrase inhibitor (Diamox 250 mg 1 x 1) was prescribed for 3 days. The intraocular pressure (IOP) decreased postoperatively in both eyes after 1 month to 16‐19 mm Hg. Best corrected distance visual acuity (CDVA) increased to 0.5 (logMAR) right eye, and 0.7 (logMAR) left eye with a binocular CDVA of 0.4 (logMAR). Best corrected near visual acuity (CNVA) increased too, but needed 3 months, most probably due to the neuroadaptation.

Four weeks postoperatively, fundoscopy and OCT examinations showed nearly unchanged conditions of the macula with subretinal hemorrhage and edema in the left eye. The cystic edema of the left macula disappeared. Therefore, another intravitreal injection of Aflibercept in the left eye was performed 6 weeks after surgery. Twelve months after surgery, the slit lamp examination showed two clear intraocular lenses in loco, an intraocular pressure of 17/16 mm Hg without any medication, stable dry ARMD conditions in the right eye and a slightly better OCT in the wet ARMD left eye. Binocular CDVA for distance was 0.5 (logMAR), binocular CNVA reached 0.92 (logMAR). Quality of life of the patient had improved significantly, she could again perform daily tasks. On a scale 0‐10, with 10 indicative of best autonomy and 0 indicative of the worst, the patient achieved a score of 3 prior to surgery and a score of 6 postoperatively. Control examinations, including OCT, were performed every 6 weeks without further IVOM injections, and no worsening of the macula was observed during the following 8 months.

## 3 DISCUSSION

The new foldable one‐piece high‐add IOL LENTIS® MAX LS‐313 MF80 is made of a copolymer acrylate, consisting of hydrophilic acrylates with a hydrophobic surface (Hydrosmart Copolymer, water content 25%). The UV absorbing lens has a 360‐degree square edge technology (optic and haptic) for posterior capsule opacification (PCO) prevention and can be implanted through a 2.2 mm clear cornea incision. The plate‐haptic IOL has an overall diameter of 11 mm and an optic diameter of 6 mm. The new lens is a further development of the asymmetric, sectorial, bifocal Lentis LS‐313 MF 30 (Oculentis) The new feature of this “high‐add” intraocular lens is a second additional near segment on the posterior surface of the lens. The sectorial bifocal acrylic lens has an aspheric biconvex design with an add power of 8.0 D, equating to 6.0 D at the spectacle plane. This corresponds to a 1.5× magnification at a distance of 30 cm and a 3× magnification at 15 cm.

**Figure 3:**
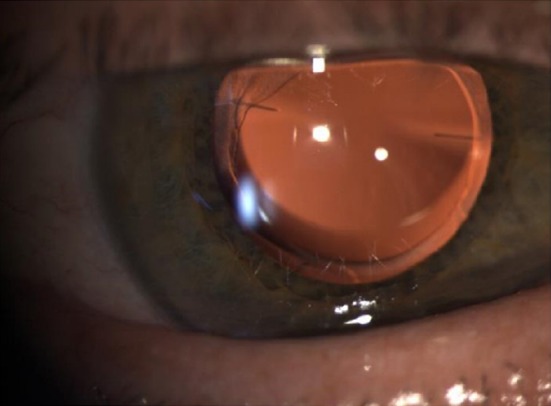
Slit lamp examination showing Lentis LS‐313 MF80 well‐positioned without PCO, 15 mo after surgery

The major benefit of a magnifying lens or magnifying surgery (MAGS) is the restoration of the ability of the patient to cope with daily life and to perform everyday activities, such as reading, viewing photos, eating, cooking, cutting nails, taking care of personal hygiene, or using a phone. Additionally, after removing the cloudy lens, the peripheral visual field improves, and the patients benefit from more contrast sensitivity, less glare, and better color perception. Due to the sector‐shaped design of the lens halos are not expected and were not reported in our case (Figure 3). The postoperative fundoscopy and OCT revealed better image quality because of the clear IOL. As described in this case report, quality of life improved almost immediately after surgery. It is important to inform the patient about the slow process of recovery for near vision due to the neuroadaptation. Patients need to be advised to train eye movements and coordination after such surgery.

There are other implantable devices for ARMD, used in implantable miniature microscope (IMT) lenses, the IOL‐VIP System, and iolAMD as a Galilean type telescope. For the Galilean approach, two optical elements with high positive and negative power are used. IMT lenses can achieve higher magnification than IOL‐VIP System and iolAMD because the positive and negative lenses are embedded in air. This configuration requires the implantation of a long tube through a larger corneal incision. Another telescope approach is the LMI, based on a Cassegrain configuration, which uses mirrors instead of lenses. It can provide high magnification, but due to the sophistication of the device it requires higher manufacturing costs, especially compared to a simple silicon or acrylic IOL. Another approach used in the Scharioth Macula Lens is based on magnification at closer distances.^12^ The closer the object to the eye, the higher the magnification. In this approach, it needs to be considered that the subject is unable to accommodate and for that reason it incorporates a + 10 D central area in the lens. Magnification is only achieved when the object is in a range of 10‐15 cm from the eye.^13^ Finally, a very promising new approach is the eyemax mono IOL (LEH Pharma, London, UK), which offers a new management of patients with ARMD undergoing cataract surgery. A single piece, soft, hydrophobic acrylic monofocal IOL was refined to generate transverse asphericity and maintain a breadth of focus across the macula (in the same way that longitudinal asphericity is employed to generate depth of focus and reduce glasses dependence). The effect is to provide a high‐quality image in all areas extending up to 10 degrees from its center where photoreceptor cell densities may still afford good visual acuities. It is termed to be a new class of “extended macular vision” IOL designed to optimize the image supplied to all areas of the macula and not just the foveal center.

Until today, some ophthalmologists believe that cataract surgery is a contraindication in late‐stage ARMD. With our study, we would like to contribute to a mind change, in that patients might benefit from new technologies, such as high‐add IOLs and from new procedures, such as combined cataract surgery and intravitreal injection. Our case report demonstrates that visual acuity and quality of life can be improved to enable patients to carry out their daily tasks independently. The increased life expectancy will irrevocably lead to a higher incidence of late‐stage ARMD and advanced cataract formation in the next years and we need to be prepared to help these patients.

We believe major opportunities arise from a new subcategory in cataract surgery—called magnifying surgery (MAGS). Our case report proves the potential of this new technology. More studies with higher number of cases should be published and we expect our long‐term results soon.^14^

## CONFLICT OF INTEREST

None declared.

## AUTHOR CONTRIBUTION

BA: performed surgery, drafted the manuscript, reviewed and approved the final version for publication. BEM: drafted the manuscript, reviewed and approved the final version for publication.
